# The Influence of MOQI APRNs on the COVID-19 Response in Nursing Homes

**DOI:** 10.1093/geroni/igab046.2123

**Published:** 2021-12-17

**Authors:** Lori Popejoy, Megan Hiltz, Marilyn Rantz, Amy Vogelsmeier

**Affiliations:** University of Missouri - Columbia, Columbia, Missouri, United States

## Abstract

During the COVID-19 pandemic Missouri Quality Initiative APRNs worked in 16 nursing homes (NHs) providing clinical expertise and support. To understand their influence on the NH COVID-19 response, we conducted four group interviews with APRNs from 13 of the 16 NHs. Using thematic analysis, we identified similarities and differences between NH groups and then compared groups by COVID-19 infection rates. Leaders from NHs with high COVID-19 rates were unwilling to report infections and were resistant to resident/staff testing. In contrast, leaders from NHs with low COVID-19 rates were strategic about acquiring supplies, held daily huddles, and initiated CDC recommendations almost immediately. All reported residents lost weight, and experienced mood and physical decline resulting from quarantine/isolation. APRNs worked with providers to identify potentially ill residents/staff, improve isolation/quarantine procedures, manage ill residents, and supported efforts to mitigate viral spread. We will discuss implications for broader infection prevention in NHs.

